# A comparative study of automatic image segmentation algorithms for target tracking in MR‐IGRT

**DOI:** 10.1120/jacmp.v17i2.5820

**Published:** 2016-03-08

**Authors:** Yuan Feng, Iwan Kawrakow, Jeff Olsen, Parag J. Parikh, Camille Noel, Omar Wooten, Dongsu Du, Sasa Mutic, Yanle Hu

**Affiliations:** ^1^ School of Mechanical and Electronic Engineering Soochow University Suzhou Jiangsu China; ^2^ Department of Radiation Oncology Washington University School of Medicine St. Louis MO USA; ^3^ Institute of Computational Engineering and Science The University of Texas at Austin Austin TX USA; ^4^ ViewRay Inc. Oakwood Village OH USA; ^5^ Department of Radiation Oncology Mayo Clinic in Arizona Phoenix AZ USA

**Keywords:** image segmentation, image‐guided radiotherapy, MRI, motion images

## Abstract

On‐board magnetic resonance (MR) image guidance during radiation therapy offers the potential for more accurate treatment delivery. To utilize the real‐time image information, a crucial prerequisite is the ability to successfully segment and track regions of interest (ROI). The purpose of this work is to evaluate the performance of different segmentation algorithms using motion images (4 frames per second) acquired using a MR image‐guided radiotherapy (MR‐IGRT) system. Manual contours of the kidney, bladder, duodenum, and a liver tumor by an experienced radiation oncologist were used as the ground truth for performance evaluation. Besides the manual segmentation, images were automatically segmented using thresholding, fuzzy k‐means (FKM), k‐harmonic means (KHM), and reaction‐diffusion level set evolution (RD‐LSE) algorithms, as well as the tissue tracking algorithm provided by the ViewRay treatment planning and delivery system (VR‐TPDS). The performance of the five algorithms was evaluated quantitatively by comparing with the manual segmentation using the Dice coefficient and target registration error (TRE) measured as the distance between the centroid of the manual ROI and the centroid of the automatically segmented ROI. All methods were able to successfully segment the bladder and the kidney, but only FKM, KHM, and VR‐TPDS were able to segment the liver tumor and the duodenum. The performance of the thresholding, FKM, KHM, and RD‐LSE algorithms degraded as the local image contrast decreased, whereas the performance of the VP‐TPDS method was nearly independent of local image contrast due to the reference registration algorithm. For segmenting high‐contrast images (i.e., kidney), the thresholding method provided the best speed (<1 ms) with a satisfying accuracy (Dice=0.95). When the image contrast was low, the VR‐TPDS method had the best automatic contour. Results suggest an image quality determination procedure before segmentation and a combination of different methods for optimal segmentation with the on‐board MR‐IGRT system.

PACS number(s): 87.57.nm, 87.57.N‐, 87.61.Tg

## I. INTRODUCTION

The advent of magnetic resonance (MR) image‐guided radiotherapy (IGRT), or MR‐IGRT, has important influences on the radiation treatment.^(1)^ The primary advantages of MR‐IGRT over existing image‐guided radiotherapy systems based on planar X‐ray imaging or cone‐beam computed tomography (CBCT) are superior soft‐tissue contrast, no radiation dose, capability of tracking tissue during radiation delivery, and multiplanar imaging. Several MR‐IGRT systems that are being investigated include MR/linac systems,^2^, ^3^, ^4^, ^5^ a MR/Co‐60 system,^6^, ^7^ and systems with either mobile patient or magnet.^(1)^ Among those, the MR/Co‐60 system from the ViewRay Inc. is commercially available and already started treating patients clinically.^(8)^

By using fast imaging sequences with short echo time (TE) and repetition time (TR), the MR‐IGRT system is able to acquire planar images at a rate of several frames per second simultaneously with radiation dose delivery.^(7)^ These images enable real‐time tracking of the moving tumor and/or organs at risk (OARs), which offers various possibilities to improve treatment efficacy. For a moving target, a large treatment margin is required to provide sufficient dose coverage to the treatment target. As a result, more healthy tissues surrounding the target, especially those that are radiosensitive, will receive a substantial amount of radiation dose, causing side effects and complications. In certain cases, the side effects and complications can be severe enough to lead to poor life quality and even death. MR‐IGRT provides the potential of tracking the moving target. Knowing the precise position and shape of the treatment target during delivery would allow effective gated radiation therapy, making it feasible to reduce treatment margin and lower radiation dose to OARs without sacrificing dose coverage to the target. Clinically, this will results in fewer side effects and better treatment efficacy.

In the clinical practice of the MR‐IGRT system, tumor margins are reduced to spare surrounding healthy tissues by allowing only the beam to be turned on when the target is within a predefined region. It is also possible to gate the radiation delivery based on one or more OARs not being allowed to enter the high‐dose region, or any combination of tumor and OAR requirements. To utilize the real‐time imaging information to improve treatment efficacy, it is necessary to identify methods that can delineate regions of interest (ROIs), targets, and/or OARs, automatically and reliably.

For MR image segmentation algorithms, intensity‐based segmentation classifies voxels into different tissue types based on intensities, and no reference image is needed.^9^, ^10^ Atlas‐based segmentation transforms an established ROI on the reference image (atlas) to the image to be segmented using rigid or deformable image registration.^11^, ^12^ Although many methods in the first category were investigated for automated segmentation of MR images,^9^, ^13^ most of the published studies were based on images acquired using conventional diagnostic MR systems with a magnetic field strength of 1.0 T or greater. Also, the scan protocols used had a relatively long acquisition time tailored for high contrast needed for the diagnostic purpose. The ViewRay MR‐IGRT system (ViewRay, Cleveland, OH) utilizes a magnetic field strength of 0.35 T.^(7)^ Compared to the segmentation task using diagnostic quality MR images, automatic segmentation of targets and/or OARs using the ViewRay images is more challenging, primarily because soft‐tissue contrast and spatial resolution have to be compromised to achieve sufficient temporal resolution. In addition, most MR image segmentation studies focused on a single anatomical site, mostly brain^14^, ^15^ and cardiac tissue.^10^, ^16^, ^17^, ^18^ However, for MR‐IGRT, it is necessary to be able to segment and track a broad range of organs and tumors (e.g., bladder, kidney, duodenum, liver tumor). For atlas‐based segmentation methods, previous studies mainly focused on segmenting a single image or a relatively small set of images^19^, ^20^ (e.g., various breathing phases of a 4D CT dataset). For MR‐IGRT, we need to be able to segment a large number of images for the purpose of gating radiation dose delivery.

In this study, we evaluated the performance of four intensity‐based segmentation methods and one atlas‐based segmentation method (provided by the ViewRay treatment planning and delivery system (VR‐TPDS)). MR motion images used in the performance evaluation were acquired using the ViewRay MR‐IGRT system for four representative clinical scenarios, with ROIs manually outlined by an experienced radiation oncologist. Metrics commonly used to quantify segmentation accuracy were included to assess the capability of each method in segmenting a single image frame, as well as motion image series.

## II. MATERIALS AND METHODS

### A. Clinical datasets

The images used in this study were acquired on the ViewRay MR‐IGRT system using the TrueFISP (true fast imaging with steady‐state free precession) sequence. Three patients (S1, S2, and S3), diagnosed with cancer in the abdominal or pelvis area, were included in this institutional review board (IRB)‐approved, imaging‐only study. For each patient, a total of 40 image frames from a series of motion tracking images that spanned up to 3 breathing cycles were selected for evaluation of the segmentation methods.

In this work, the bladder, kidney, duodenum, and the liver tumor were selected to evaluate segmentation algorithms. This is because the bladder is usually considered an organ of interest in the treatment of bladder or prostate cancer. The moving kidney is a known challenge in treating adjacent tumors. The duodenum is considered an organ of interest in the treatment of liver tumors. A liver tumor was selected because it was directly susceptible to respiration‐induced motion.^(21)^ Due to the irregular nature of respiration, variation in respiration‐induced organ motion may occur between breathing cycles. Hence, it is necessary to evaluate segmentation algorithms through multiple breathing cycles. Meanwhile, we need to limit the number of images to keep the time needed for the manual segmentation within an acceptable level. In this study, 40 consecutive frames covering up to 3 respiratory cycles were selected, which provided a sufficient number of images for performance evaluation and statistical analysis.

**Figure 1:**
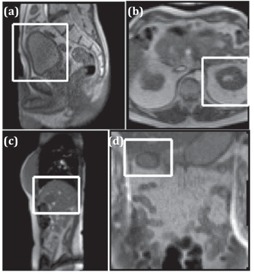
Sample motion images containing (a) a sagittal view of bladder (S1), (b) an axial view of kidney (S2), (c) a sagittal view of liver tumor (S3), and(d) a coronal view of duodenum (S2). The white box indicates the reduced field of view (FOV) used in the segmentation.

**Figure 2:**
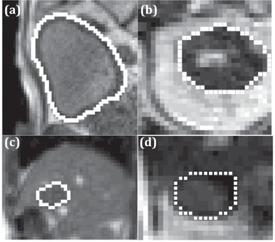
Sample radiation oncologist's manual segmentations (white line) of the (a) bladder, (b) kidney, (c) liver tumor, and (d) duodenum in Fig. 1.

Figure 1 shows the ViewRay motion images of the bladder (Fig. 1(a)), kidney (Fig. 1(b)), liver tumor (Fig. 1(c)), and a section of duodenum (Fig. 1(d)). The parameters used to acquire these images are summarized in Table 1. In general, the intensity‐based segmentation methods require a reduced field of view (FOV) around the region to be segmented, whereas the atlas‐based segmentation method uses the entire image to perform deformable image registration to a reference frame. The reduced FOV used in this study is shown with the white rectangles in Fig. 1. The size of the reduced FOV was selected in such a way so that the targets were kept in the FOV throughout the motion. To evaluate the performance of the segmentation algorithms, a board‐certified academic gastrointestinal (GI) radiation oncologist's manual contours of the organs or tumor in each motion frame were used as the ground truth (Fig. 2).

**Table 1:** MR imaging parameters used to acquire the motion images

	Fig. 1(a)	Fig. 1(b)	Fig. 1(c)	Fig. 1(d)
TR (ms)	3.3	2.8	2.9	2.8
TE (ms)	1.4	1.2	1.3	1.2
FOV (${\text{mm}}^{2}$)	270×270	270×270	351×450	270×270
Slice thickness (mm)	5	7	5	7
Image matrix	110×110	78×78	100×128	78×78
Pixel size (${\text{mm}}^{2}$)	2.5×2.5	3.5×3.5	3.5×3.5	3.5×3.5
Imaging plane	Sagittal	Axial	Sagittal	Coronal

### B. Local image contrast

The image contrast is closely related to the segmentation performance. In this study, the local image contrast $L_{c}$ is defined as:^(22)^

(1) $$L_{c}=\frac{1}{n}\sum_{i=1}^{n}lc_{i}$$

where *n* is the total number of pixels in the image and ${\text{lc}}_{i}$ is the average difference of local contrast between the pixel *i* and its adjacent four‐connected neighbors:

(2) $$lc_{i}=\frac{1}{4}(\left|PC_{i}-PC_{i-1}\right|+\left|PC_{i}-PC_{i+1}\right|+\left|PC_{i}-PC_{i-col}\right|+\left|PC_{i}-PC_{i+col}\right|)$$

Here, $PC_{i}=100{\left(\frac{k}{255}\right)}^{1.1}$ is the pixel contrast at each pixel point, *k* is the pixel value, and *col* is the image resolution in the vertical direction.

### C. Segmentation methods

Among many intensity‐based and atlas‐based segmentation methods,^23^, ^24^ five algorithms were selected. The global thresholding method was chosen as a representative thresholding method. For unsupervised learning method, the fuzzy k‐means (FKM) and k‐harmonic means (KHM) were chosen. For partial differential equation‐based deformable models, we selected a modified reaction‐diffusion level‐set evolution (RD‐LSE) method. For the atlas‐based method, the VR‐TPDS was used. Detailed description of each segmentation method can be found in Appendix A. To compare all the gray‐scale images within the same level, all the images tested were linearly transformed to have 256 gray levels. For the final contouring of the objective organ, necessary morphological image processing steps were used for postprocessing. A test‐run was needed to identify the cluster group (FKM and KHM), the range below or above the threshold (thresholding), and the appropriate level set (RD‐LSE). The image segmentation was performed using custom codes written in MATLAB (MathWorks, Natick, MA).

### D. Segmentation evaluation

#### D.1 Segmentation evaluation metrics

Although many methods have been proposed for segmentation performance evaluation,^25^, ^26^, ^27^, ^28^ detecting the correct geometry is of primary concern in accurate motion tracking of the target. In this study, Dice coefficient and target registration error (TRE) were used to quantitatively evaluate the performance of the segmentation methods.

Given the segmented structure as A, the ground truth structure as B, and |*| representing the size of a binary set, the Dice coefficient^(29)^ $C_{\text{dic}}$ is defined as

(3) $$C_{dic}=\frac{2\left|A\cap B\right|}{\left|A\right|+\left|B\right|}.$$

The Dice coefficient represents the ratio of overlapped region between the segmented region and the truth region ($0\leq C_{dic}\leq 1$). The maximum value of $C_{\text{dic}}$ is 1 when the segmented region is identical with the truth region, and the minimum value is 0 when the segmented region totally misses the truth region.

Given that the centroid position of the manually outlined ROI is ($x_{\text{man}}$, $y_{\text{man}}$) and the centroid position of the automatically segmented ROI is ($x_{\text{seg}}$, $y_{\text{seg}}$), the TRE is defined as

(4) $$TRE=\sqrt{{\left(x_{man}-x_{seg}\right)}^{2}+{\left(y_{man}-y_{seg}\right)}^{2}}$$

#### D.2 Statistical analysis

To evaluate the performance difference, a Student's *t*‐test for two independent samples was used by assuming a normal distribution for the obtained metric values. The p‐value was computed using the metric values obtained from the 40 images. The number of data points was sufficiently large to assume a normal distribution for the obtained metric average for each algorithm and, therefore, the use of the Student's *t*‐test was justified. A significance level of 5% was considered to show a statistically significant difference between the performances of two algorithms for a given metric.

## III. RESULTS

### A. Local image contrast

**Figure 3:**
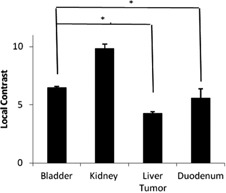
Mean and standard deviation of local contrast. The contrast values of bladder and kidney are significantly higher than that of liver tumor and duodenum (p<0.01). The horizontal bars indicate a comparison between two different targets. The asterisks show a significant contrast difference between the two targets.

The distribution of the local image contrast for each organ or tumor is shown in Fig. 3. The local contrast of the kidney and the bladder is significantly higher than that of the liver tumor and the duodenum.

### B. Segmentation of a single image frame

**Figure 4:**
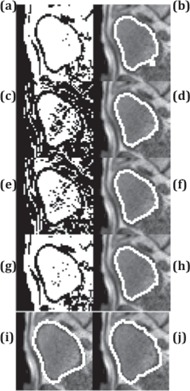
Comparison of the bladder segmentation of a sample image frame using five different methods. The left column contains the original segmentation results based on (a) global thresholding, (c) FKM, (e) KHM, and (g) RD‐LSE. In the right column are the corresponding final segmentation results ((b), (d), (f), (h)) after morphological processing. (i) is the first frame with manual contour as a reference where (j) is the deformable registered contour based on VR‐TPDS algorithm.

**Figure 5:**
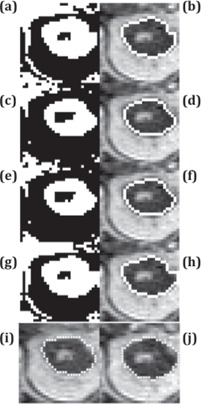
Kidney segmentation of a sample image frame using five different methods. The left column contains the original segmentation results based on (a) global thresholding, (c) FKM, (e) KHM, and (g) RD‐LSE. In the right column are the corresponding final segmentation results ((b), (d), (f), (h)) after morphological processing. (i) is the first frame with manual contour as a reference where (j) is the deformable registered contour based on VR‐TPDS algorithm.

**Figure 6:**
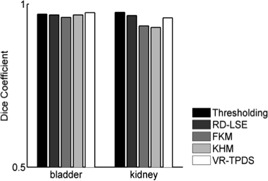
Dice coefficient from segmentation of a single image frame of bladder and kidney.

**Figure 7:**
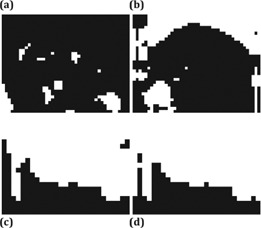
Segmentation results of the ((a) (b)) liver tumor and ((c), (d)) duodenum based on ((a), (c)) global thresholding, and ((b), (d)) RD‐LSE. The two methods could not provide a recognizable target region.

**Figure 8:**
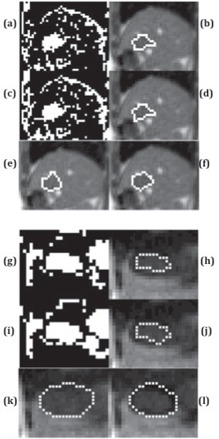
Segmentation of the liver tumor based on FKM ((a) (b)) and KHM ((c), (d)); and segmentation of the duodenum using FKM ((g), (h)) and KHM ((i), (j)). The left column shows the original segmentation results while the right column shows the final results after morphological processing. ((e), (k)) is the first frame with manual contour as a reference where ((f), (i)) is the deformable registered contour based on the VR‐TPDS algorithm.

In the segmentation of a single image frame, all methods successfully segmented the bladder (Fig. 4) and the kidney (Fig. 5). The Dice coefficient of all methods was close to each other (Fig. 6). Thresholding and RD‐LSE could not provide a recognizable target contour when segmenting the liver tumor and the duodenum (Fig. 7), which had a lower local contrast compared to the bladder and kidney (Fig. 3). The FKM, KHM and VR‐TPDS methods successfully segmented the target region (Fig. 8), despite the lower local contrast.

### C. Segmentation of motion image series

**Figure 9:**
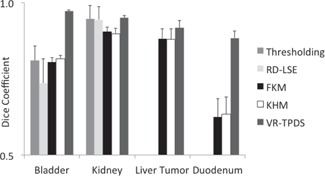
Comparison of Dice coefficient of the five segmentation methods in segmenting bladder, kidney, liver tumor, and duodenum. The segmentation is with respect to the 40 motion image frames of each target.

The evaluation of the segmentation of motion image series was separated into two categories, images with relatively high contrast (bladder and kidney) and with relatively low local contrast (liver tumor and duodenum). For each target, the Dice coefficient was evaluated at each of the 40 motion image frames. Based on that, the mean value and standard deviation of the Dice coefficient were calculated and compared among all methods (Fig. 9). The corresponding statistical significance was also summarized (Table 2). Note that when the p‐value is not explicitly given in the text, it can be found in Table 2, which lists the p‐values of all investigated algorithm pairs for the four targets studied in this paper.

For segmentation of the bladder, no significant differences in the Dice coefficient were found between the thresholding (0.81), FKM (0.80), and KHM methods (0.82). The Dice coefficient of RD‐LSE was 0.74, which was the lowest among all methods (this result was statistically significant, see Table 2). The average Dice coefficient of VR‐TPDS was 0.97; this was significantly higher than all other methods. The standard deviation for RD‐LSE had the highest value. This indicated that the RD‐LSE method tended to have a larger segmentation variation with a high error rate.

For segmentation of the kidney, results show that the Dice coefficients for thresholding, RD‐LSE, and VR‐TPDS were significantly higher than that of FKM and KHM. No significant differences of the Dice coefficient were found between thresholding, RD‐LSE, and VR‐TPDS. FKM and KHM also yielded similar performance with no significant differences; thresholding and RD‐LSE also showed to have very similar performance. The standard deviations for thresholding and RD‐LSE were equal or higher than those of FKM, KHM, and VR‐TPDS.

**Table 2:** P‐values of the Dice coefficients. The pairwise Student's *t*‐tests were between different algorithms for each target. P‐values were rounded to two significant digits, so a value of zero indicated a p‐value less than 0.005 and a significant difference between the two methods compared

	*Bladder*	*Kidney*	*Liver Tumor*	*Duodenum*
FKM vs. KHM	0	0.11	0.69	0.51
FKM vs. VR‐TPDS	0	0	0	0
KHM vs. VR‐TPDS	0	0	0	0
Thresholding vs. RD‐LSE	0	0.78		
Thresholding vs. FKM	0.51	0		
Thresholding vs. KHM	0.37	0		
Thresholding vs. VR‐TPDS	0	0.63		
RD‐LSE vs. FKM	0	0		
RD‐LSE vs. KHM	0	0		
RD‐LSE vs. VR‐TPDS	0	0.38		

For the segmentation of the liver tumor and duodenum, only FKM, KHM, and VR‐TPDS could segment the target region (Fig. 8). For the liver tumor, the mean Dice coefficients of FKM and KHM were 0.88, while VR‐TPDS had a mean Dice coefficient of 0.92. For the duodenum, the average Dice coefficient was 0.63 for KHM and FKM, and 0.88 for VR‐TPDS. The differences between FKM and KHM were not statistically significant.

**Figure 10:**
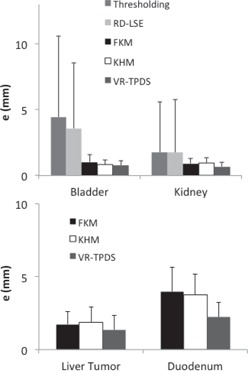
Comparison of TRE values (in mm) between the centroid of the automatically segmented targets and the manually segmented targets for (a) bladder and kidney, (b) liver tumor and duodenum.

**Figure 11:**
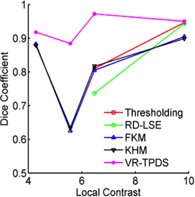
Mean Dice coefficient vs. local contrast values for each of the segmentation algorithms.

To demonstrate the accuracy of target tracking from real‐time MR planar images, we compared the TRE value of each method (Fig. 10). The mean TRE values of thresholding and RD‐LSE for tracking bladder were 4.45 mm and 3.58 mm, for kidney were 1.76 mm and 1.75 mm, respectively. The mean TRE values of FKM and KHM are very similar: 1.04 mm vs. 0.81 mm for bladder, 0.92 vs. 0.96 mm for kidney, 1.72 vs. 1.87 mm for the liver tumor, and 3.94 vs. 3.57 mm for the duodenum. The VR‐TPDS method had TRE values of 0.78 mm, 0.69 mm, 1.34 mm, and 2.21 mm for the bladder, kidney, liver tumor, and duodenum. We compared the p‐values between FKM, KHM, and VR‐TPDS methods since thresholding and RD‐LSE had large TRE values (Table 3). A comparison of the Dice coefficient vs. local contrast value for each segmentation method is shown in Fig. 11.

**Table 3:** P‐values of the TRE values. The Student's *t*‐tests were between FKM, KHM, and VR‐TPDS for different targets. P‐values were rounded to two significant digits, so a value of zero indicated a p‐value less than 0.005. The “*” symbol indicates a significant difference between the two methods

	*Bladder*	*Kidney*	*Liver tumor*	*Duodenum*
FKM vs. KHM	0.04*	0.68	0.50	0.58
FKM vs. VR‐TPDS	0.01*	0*	0.02*	0*
KHM vs. VR‐TPDS	0.37	0*	0*	0*

## IV. DISCUSSION

Thresholding is the easiest method to implement and has the fastest processing speed. When segmenting the kidney, it did not show a difference in Dice coefficient compared to RD‐LSE and VR‐TPDS. This indicates that, if the image to be segmented has a high contrast value, the straightforward thresholding method can provide a satisfying automatic contour with the best speed. This is desirable in the real‐time MR‐IGRT. However, the thresholding method is very sensitive to the image contrast. When the image contrast was getting lower, as in the bladder case, the Dice coefficient reduced to 0.81. In the case of liver tumor and duodenum, where the local contrast value was below 6, thresholding failed to segment out the target region. The performance of RD‐LSE was similar to thresholding; however, the RD‐LSE suffered from prolonged processing time that is not desirable in real‐time MR‐IGRT.

FKM, KHM, and VR‐TPDS successfully segmented all the target regions. However, in segmenting kidney, FKM and KHM methods had lower Dice coefficients compared with other methods. In segmenting the bladder, the Dice coefficients of FKM and KHM were lower than that of VR‐TPDS. This was because the contrast was different between the fluid‐filled region and the bladder wall. The FKM and KHM tended to segment only the fluid‐filled region of the bladder but not the bladder wall. By comparing the TRE value, KHM was not significantly different from VR‐TPDS (Table 3). In segmenting the liver tumor and duodenum, FKM and KHM showed no significant difference (Table 2, Table 3).

Although the local contrast was higher in the motion images of the duodenum than those of the liver tumor, evaluation metrics were better in segmenting the liver tumor than the duodenum (Fig. 11). This was primarily due to the fact that the contrast distribution was different. In the duodenum, most of the contrast was concentrated at the lower part of the duodenum region, while the upper part of the duodenum had low contrast. The liver tumor had a relatively uniform distribution of contrast along its boundary. In segmenting liver tumor and duodenum, VR‐TPDS had a significant higher value of Dice coefficient and TRE. The results indicate that VR‐TPDS method is more suitable in segmenting images with low contrast.

It is necessary to select a reduced FOV for automatic segmentation for the intensity‐based methods. This allows the object organ to possess major voxel information in the analysis, and also reduces the processing time. Similarly, the reduced FOV have been used in segmentation of positron emission tomography (PET) images.^(30)^ When segmenting motion image series, it is required that the reduced FOV to include the object organ for all motion image frames. While the reduced FOV is required for the thresholding, RD‐LSE, FKM, and KHM methods, a reference image is needed for the VR‐TPDS algorithm to initialize deformable registration.

Although an additional manual input to contour the target region as a reference is needed, it provides the advantage of tracking nonorgan contours.

The performance of all the reference‐free methods is greatly influenced by image contrast. For the kidney region that has the highest average local contrast value, FKM and KHM have the lowest Dice coefficient value (Fig. 9). However, all the other methods showed no significant difference (Table 2). As the local image contrast decreases (e.g., in the images of the bladder), the values of the Dice coefficient of thresholding and RD‐LSE become lower than those of FKM and KHM (Fig. 9). When the average local contrast drops below 6 (liver tumor and duodenum), only FKM and KHM can successfully segment the target region. Contrary to the intensity‐based methods, the atlas‐based method (VR‐TPDS) is relatively contrast‐insensitive and can successfully segment the target region even when the average local contrast drops below 6. This is because the VR‐TPDS method is based on a deformable registration algorithm. The segmentation is based on the overall image contrast and the availability of the reference image. It is not based on pixel‐wise calculation as other methods and, therefore, is relatively insensitive to local image contrast. However, as the image contrast decreases, the segmentation performance was impaired.

Some segmentation studies use certain pre‐processing procedures before segmentation. The preprocessing filters commonly used in the literature include Gaussian filter,^(30)^ nonlinear anisotropic smoothing filters,^(16)^ adaptive anisotropic filter,^(31)^ bilateral filter.^(32)^. The main purpose of applying filtering before segmentation is to reduce the noise in the image, especially for images with low signal‐to‐noise ratio. Although applying filtering before segmentation tends to improve the overall segmentation results, it is noted that different filtering methods may only work with different segmentation methods. In this study, in order to compare the segmentation methods on an equal stage, we focused on the segmentation of original MR images without filtering. Although postprocessing of segmented images may not be necessary in algorithm study of synthetic images, it is a necessary step to get desirable contours to process the segmented images in clinical application. Commonly used postprocessing methods involve morphological processing such as region filling and extraction of connected components. Since the postprocessing procedures affect the final contouring results, the procedure was kept as consistent as possible to minimize the influence. No postprocessing steps were needed for the VR‐TPDS method.

In the evaluation of segmentation methods, prior studies have used many metrics^26^, ^27^ such as performance metrics,^(25)^ relative ultimate measurement accuracy,(28) and similarity coefficient.^(17)^ Hoover et al.^(25)^ introduced performance metrics based on different classification of the region. Zhang^(28)^ proposed a relative ultimate measurement accuracy based on image feature. Other metrics similar to Dice coefficient, such as the similarity coefficient,^(17)^ was also used. The focus of this study is to evaluate the segmentation performance of the various algorithms for the purpose of real‐time target tracking during radiation therapy delivery. In this context, a high value of the Dice coefficient, which captures the accuracy of the target delineation in different ways, is important for precise treatment delivery. At the same time, the TRE value that captures the overall position of the target ROI is important for the control of radiotherapy delivery. Manual contours have been serving as ground truth as an established practice — for example in segmenting brain tumor,^(33)^ hippocampus region,^(34)^ esophageal and gastroesophageal cancer,^(35)^ among others. Although there is variability in physician's contour of regions of interest, it is a known limitation of studies evaluating efficacy of segmentation algorithms in medical imaging.^(36)^ The appropriateness of using manual contours to serve as ground truth warrants a separate investigation and is beyond the scope of this manuscript.

Current marker based radiotherapy tracking methods are based on monitoring one or more points defined by fiducial markers or electromagnetic transducers. Tracking can be direct via transmission X‐ray imaging or directly determining the position of the electromagnetic emitters, or indirect via correlation models to a surrogate such as the patient surface. Similar to the error measurement of TRE, point tracking methods are believed to achieve accuracy in the range of 2 mm.^37^, ^38^, ^39^ A recent method using 1D MRI pencil‐beam navigators to track tissue motion reported accuracy within 1.5 mm in one case of kidney motion.^(40)^ Another real‐time markerless motion tracking method for lung tumor tracking showed a root mean square deviation less than 1 mm.^(41)^ Here we showed that the tracking error was also within 1 mm when using FKM, KHM, and VR‐TPDS methods for segmenting bladder and kidney. However, when segmenting the liver tumor and duodenum region that had a lower image contrast, the largest tracking error could be 4 mm. In the current MR‐IGRT system, the overlapping of a predefined region and the segmented target in motion controls the radiation beam. Thus, a larger TRE value would result in a larger position deviation of the target from the predefined region, putting the surrounding healthy region at risk.

## V. CONCLUSIONS

In this study, five different segmentation methods were compared using clinical MR images from a Co‐60 MR‐IGRT system. Segmentation performances were evaluated by comparing to manually outlined targets using Dice coefficient and TRE. The performance of thresholding, RD‐LSE, FKM, and KHM methods were closely related to local image contrast, decreasing as contrast became lower. The VR‐TPDS algorithm was less sensitive to local contrast. However, the method required a reference image with an outline of the target. The other four methods did not require a reference frame, but needed a reduced FOV that fully captures the range of target motion to improve performance. In segmenting images with a high contrast (e.g., kidney in this study), thresholding had the best speed with a satisfying accuracy. When the image contrast was low, VR‐TPDS had the best automatic contour. Given the complexity of cancer treatment, there may not exist one algorithm suitable for all target regions. To achieve the best results, variables like local contrast, size of ROI, and presence of the reference image need to be considered in the selection of an appropriate algorithm. Based on the results of this study, the thresholding method provides a fast segmentation with satisfying accuracy for images with a high contrast. For images with low contrast, it is better to use VR‐TPDS method. The results also suggest adding preprocessing steps to assess image quality and determine the optimal segmentation method. Future studies include a selection of optimal segmentation methods based on image/organ‐specific information, different filtering methods and their influences on the segmentation results, and evaluations of performance for additional organs and targets of interest in radiation therapy.

## ACKNOWLEDGMENTS

Support from grant BK20140356 from Jiangsu Province and grant 61503267 from Natural Science Foundation of China are acknowledged.

## COPYRIGHT

This work is licensed under a Creative Commons Attribution 4.0 International License.

## APPENDICES

### Appendix A. Details of the Segmentation Algorithms

#### A. Global thresholding

A discriminate analysis based method,^42^, ^43^ also known as Otsu's method, uses an optimum threshold value $T^{*}$. $T^{*}$ is determined by maximizing a discriminate criterion, or equivalently, the variance $\sigma_{B}^{2}$:

(A.1) $$\sigma_{B}^{2}(T)=\frac{{\left(\mu_{T}\omega (T)-\mu (T)\right)}^{2}}{\omega (T)(1-\omega (T))}$$

where $\sigma_{B}^{2}$ is a function of threshold value *T*, ō(*T*) and μ(*T*) are the zeroth and first‐order cumulative moments of the histogram up to the *T*th pixel value, and $\mu_{T}$ is the total first‐order cumulative moment or the total mean level of the image. A typical run time for the thresholding algorithm was less than 1 ms for a 2D image with 50×50 pixels.

### B. Fuzzy k‐means (FKM) clustering

The fuzzy k‐means (FKM, or fuzzy c‐means) clustering method is an unsupervised method^23^, ^44^ that uses a soft membership function

(A.2) $$m(i,j)=\frac{{\left\|x_{i}-c_{j}\right\|}^{-\frac{2}{r-1}}}{\sum_{j=1}^{k}{\left\|x_{i}-c_{j}\right\|}^{-\frac{2}{r-1}}},$$

where $x_{i}$ is the value of each pixel, $c_{j}$ is centroid of each cluster group, *k* is the total number of cluster groups, ‖■‖ is the Euclidean distance, and *r* is taken as 2. Pixels are grouped into the closest clusters by the membership function. The cluster centroid value, $c_{j}$, is updated by iteration:

(A.3) $$c_{j}=\frac{\sum_{i=1}^{n}m(i,j)w_{i}x_{i}}{\sum_{i=1}^{n}m(i,j)w_{i}}.$$

Here, *n* is the total number of pixels and $w_{i}$ is a weighting number taken as 1. In this study, four clustering groups are used, except in the case of duodenum where three groups are used. The initial centroid pixel values were chosen to span the image intensity value equally. The FKM performance function is defined as:

(A.4) $$J_{FKM}=\sum_{i=1}^{n}{\sum_{j=1}^{k}m{\left(i,j\right)}^{r}\left\|x_{i}-c_{j}\right\|}^{2},$$

where *n* is the total number of pixels. The iteration ends when the change of $J_{\text{FKM}}$ is within $10^{-6}$. In the segmentation of the liver tumor and the duodenum, where the image contrast is low, a histogram equalization step was applied before the segmentation. At the end of the iterative clustering, the pixels are assigned to belong to the cluster where m(i,j) has a maximum. A cluster number obtained from a test‐run selects the specific pixel group containing the segmentation target. A typical run time for the FKM algorithm was about 10 ms for a 2D image with 50×50 pixels.

### C. K‐harmonic means (KHM) clustering

Similar to FKM, K‐harmonic means (KHM) clustering^(45)^ has a membership function as:

(A.5) $$m(i,j)=\frac{{\left\|x_{i}-c_{j}\right\|}^{-p-2}}{\sum_{j=1}^{k}{\left\|x_{i}-c_{j}\right\|}^{-p-2}},$$

where $x_{i}$ is the value of each pixel, $c_{j}$ is the centroid of each cluster group, *k* is the total number of cluster group, ‖■‖ is the Euclidean distance and *p* is taken as 2. The centroids are updated within each iteration loop according to Eq. (A.4), except that the weighting function is different:

(A.6) $$w(i)=\frac{\sum_{j=1}^{k}{\left\|x_{i}-c_{j}\right\|}^{-p-2}}{{\left(\sum_{j=1}^{k}{\left\|x_{i}-c_{j}\right\|}^{-p}\right)}^{2}}.$$

The performance function $J_{\text{KHM}}$ is:

(A.7) $$J_{KHM}=\sum_{i=1}^{n}\frac{k}{\sum_{j=1}^{k}{\left\|x_{i}-c_{j}\right\|}^{-p}},$$

where *n* is the total number of pixels and *p* is taken as 2. The initial centroid values were chosen to span the image intensity value equally. When implementing the KHM algorithm, a small positive value $\varepsilon =10^{-6}$ is used to replace the denominator in the above equations when the pixel value is the same as the centroid value. The iteration is set to end when the change of the KHM performance function is within $10^{-6}$. A histogram equalization was also applied if the image contrast was low. A typical run time for the KHM algorithm was about 100 ms for a 2D image with 50×50 pixels.

### D. Level set evolution (LSE) method

A modified reaction‐diffusion (RD) level set evolution (LSE) method (RD‐LSE)^46^, ^47^ uses a partial differential equation:

(A.8) $$\frac{\partial \phi }{\partial t}=\varepsilon \Delta \phi +\frac{F}{\varepsilon }\left|\nabla \phi \right|,$$

where *F* is a function proportional to the curvature of ϕ, and ∇ is the gradient operator, and Δ is the Laplace operator. A total of 600 iterations were used and time steps for evolution and diffusion were set to 0.01 and 0.001, respectively. A typical run time for the RD‐LSE algorithm was about 800 ms for a 2D image with 50×50 pixels. This is significantly longer than the frame rate of the imaging system (250 ms), so a practical application would require additional work to optimize the performance of this algorithm.

### E. The VR‐TPDS algorithm

The ViewRay TPDS (VR‐TPDS) method requires a template to perform the automatic segmentation. During tracking, each new image is matched to the template using a deformable image registration (DIR) algorithm. The deformation vector field obtained in this way is then used to transform the known contour of the region of interest (ROI) on the template to the current image. It is possible to deform any number of ROIs outlined on the key frame to the current image. The DIR algorithm minimizes a cost function, which consists of one minus the correlation coefficient of the current image and the key frame plus a term proportional to the sum of the squares of the gradients of the vector deformation field:

(A.9) $$\begin{array}{l} F(A,B*)=1-\frac{N\sum A_{i}B_{i}^{*}-\sum A_{i}\sum B_{i}^{*}}{{\left[(N\sum A_{i}^{2}-{\left(\sum A_{i}\right)}^{2})(N\sum B_{i}^{{*}^{2}}-{\left(\sum B_{i}^{*}\right)}^{2})\right]}^{\frac{1}{2}}}\\ \;\;\;\;\;\;+\frac{\lambda }{N}\left[\sum {\left(\frac{\partial u_{i}}{\partial x}\right)}^{2}+\sum {\left(\frac{\partial u_{i}}{\partial y}\right)}^{2}+\sum {\left(\frac{\partial v_{i}}{\partial x}\right)}^{2}+\sum {\left(\frac{\partial v_{i}}{\partial y}\right)}^{2}\right], \end{array}$$

Here, $A_{i}$ is the image intensity of the key frame image in pixel *i* at location ($x_{i}$, $y_{i}$), $B_{i}^{*}$ is, the image intensity of the current image at location $\left(x_{i}+u_{i},y_{j}+v_{i}),(u_{i},v_{i}\right)$ is the 2D deformation vector of voxel *i, N* is the number of voxels, λ is an adjustable parameter, and the sums run over all pixels of the images. The solution of the optimization problem is obtained as the solution of the corresponding Euler‐Lagrange differential equations

(A.10) $$\lambda \Delta u_{i}+\frac{\partial F}{\partial B_{i}^{*}}\frac{\partial B_{i}^{*}}{\partial x}=0,\;\lambda \Delta v_{i}+\frac{\partial F}{\partial B_{i}^{*}}\frac{\partial B_{i}^{*}}{\partial y}=0,$$

with Δ denoting the Laplace operator. The Euler‐Lagrange equations are solved via an iterative Gauss‐Seidel technique.

To improve the efficiency, accuracy, and stability of the DIR algorithm, the following methods are used:

a) A multiscale approach is utilized; this is to say, the problem is first solved on a lower resolution and the obtained solution is used as an initial guess for the current resolution. By default, two levels are employed.

b) The “forward” and “backward” registration problems (i.e., registration of the key frame to the current motion image and the current image to the key frame) are solved simultaneously. After every iteration, the forward and backward vector deformation fields are forced to be consistent:

(A.11) $$\begin{array}{l} u^{f}(x,y)=-u^{b}(x+u^{f}(x,y),y+v^{f}(x,y)),\\ v^{f}(x,y)=-v^{b}(x+u^{f}(x,y),y+v^{f}(x,y)),\\ u^{b}(x,y)=-u^{f}(x+u^{b}(x,y),y+v^{b}(x,y)),\\ v^{b}(x,y)=-v^{f}(x+u^{b}(x,y),y+v^{b}(x,y)), \end{array}$$

where the superscripts “f” and “b” are used to indicate the “forward” and “backward” deformation fields.

c) For each cine image. three different solutions are obtained: i) a solution where the initial guess for the deformation is zero; ii) a solution starting from the vector deformation field obtained for the last motion image; iii) a solution starting from an initial guess obtained by linearly extrapolating the solutions from the last two motion images. The solution having the highest similarity (as measured by the correlation coefficient) becomes the solution for the current cine image.

A typical run time for the DIR algorithm is in the range of 20 ms for 2D planar images with 100×100 pixels using five iterations (note: the number of iterations is doubled for each lower resolution scale, so 10 iterations in the first lower resolution scale and 20 iterations for the second are used). The adjustable parameter Δ, is set to 6 for all segmentations performed in this study.

## Supporting information

Supplementary Material Files
