# Supplementary figures and images for: A comparative study of automatic image segmentation algorithms for target tracking in MR‐IGRT
Source: J Appl Clin Med Phys. 2016 Mar 8;17(2):441–60. doi: 10.1120/jacmp.v17i2.5820 (PMC5875567; doi:10.1120/jacmp.v17i2.5820)

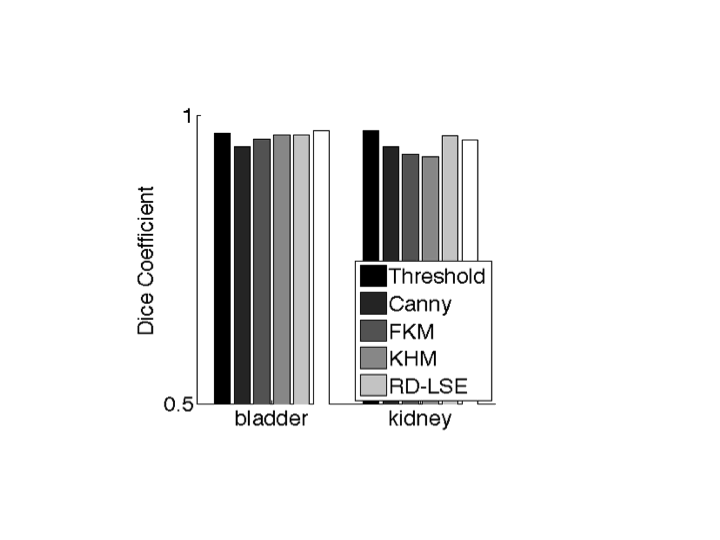

Supplement: Supplementary file 1 — Supplementary Material Files [file ACM2-17-441-s001.gif]
